# Evaluating screening for autism spectrum disorder using cluster randomization

**DOI:** 10.1038/s41598-024-57656-0

**Published:** 2024-03-21

**Authors:** Sigridur Loa Jonsdottir, Evald Saemundsen, Elin Astros Thorarinsdottir, Vilhjalmur Rafnsson

**Affiliations:** 1 State Diagnostic and Counseling Center, Dalshraun 1B, 220 Hafnarfjordur, Iceland; 2 Center of Public Health Sciences, Faculty of Medicine, University of Iceland, Reykjavík, Iceland; 3 Faculty of Medicine, University of Iceland, Reykjavík, Iceland; 4 Department of Psychology, University of Iceland, Reykjavík, Iceland; 5 Center of Children’s Mental Health, Reykjavík, Iceland; 6 Department of Preventive Medicine, Faculty of Medicine, University of Iceland, Reykjavík, Iceland

**Keywords:** Autism spectrum disorder, Screening, M-CHAT-R/F, Usual care, Cluster randomization, Diseases, Neurological disorders, Autism spectrum disorders

## Abstract

We evaluated the rate of autism spectrum disorder (ASD) in a group invited to a screening program compared to the rates in two groups who received usual care. The population eligible for screening was all children in Iceland registered for their 30-month well-child visits at primary healthcare centers (PHCs) from March 1, 2016, to October 31, 2017 (*N* = 7173). The PHCs in the capital area of Reykjavik were the units of cluster randomization. Nine PHCs were selected for intervention (invited group), while eight PHCs received usual care (control group 1). PHCs outside the capital area were without randomization (control group 2). An interdisciplinary team, including a pediatrician contributing with physical and neurological examination, a psychologist evaluating autism symptoms using a diagnostic instrument, and a social worker interviewing the parents, reached a consensus on the clinical diagnosis of ASD according to the ICD-10 diagnostic system. Children in the population were followed up for at least two years and 119 cases were identified. The overall cumulative incidence of ASD was 1.66 (95% confidence interval (CI): 1.37, 1.99). In the invited group the incidence rate was 2.13 (95% CI: 1.60, 2.78); in control group 1, the rate was 1.83 (95% CI: 1.31, 2.50); and in control group 2, the rate was 1.02 (95% CI: 0.66, 1.50). Although the rate of ASD was higher in the invited group than in the control groups, the wide confidence intervals prevented us from concluding definitively that the screening detected ASD more readily than usual care.

## Introduction

Screening for autism spectrum disorder (ASD) has been frequently applied in many countries and in different settings with the aim of an early detection^1–3^. Both the screening procedure and the screening test, for example the Modified Checklist for Autism in Toddlers, Revised with Follow-Up (M-CHAT-R/F)^4^, have been evaluated in several population-based studies^5–10^. Most of the screening studies in the literature deal solely with follow-up of the screen positive children^1–3^, but in the present study we aim to evaluate how effective screening is to detect ASD. Thus, to find out whether ASD is in fact detected earlier in a group offered screening than in a control group receiving usual care, we used randomized screening trials^11^. We are not aware of any existing studies that evaluate screening for ASD using randomization, wherein a group invited to screening is compared to a control group receiving usual care. However, a similarly designed study has been planned to include screening for ASD and a high-quality treatment with long term follow-up of outcomes^12^.

The present study is part of a project on ASD screening at the 30-month well-child visit at primary healthcare centers (PHCs) in the capital area of Reykjavik. The first phase of the project focused on the education of well-child care professionals and the implementation of ASD screening using the M-CHAT-R/F^13^. The second phase evaluated the ability of the M-CHAT-R/F to detect ASD and calculated the test’s sensitivity and specificity^14^. The aim of this population-based study, the third phase of the project, was to evaluate the rate of ASD in the group invited to the screening program in comparison with the rates in two groups who received usual care.

## Methods

This is a prospective comparison study. The population eligible for screening included all children in Iceland registered for their 30-month well-child visits at PHCs during the period from March 1, 2016, to October 31, 2017, a total of 7173 children, according to the National Registry. The National Registry is the basic register of the Icelandic population. It provides current information about Icelandic citizens and foreign citizens who are or have been domiciled in Iceland. It includes information of name, sex, birthplace, personal identity number, nationality, family relationship, and residential address. Based on the National Registry, information was obtained on the registration of an individual child to a particular PHC in the capital area. Using the National Registry, it was possible to count the children outside the capital area. The setting in the capital area of Reykjavik, Iceland, with its comprehensive health care and population registers, offers an opportunity to define and recruit populations and to assign them to different groups with the same inclusion and exclusion criteria: one which is invited to participate in a screening program for ASD while the other receives usual care. A nationwide database on diagnosed ASD cases at the State Diagnostic and Counseling Center (SDCC) ensured proper follow-up and rigorous establishing of the diagnoses without discrimination between children based on residence or ethnicity^15^.

The rationale for choosing the capital area of Reykjavik for screening, as opposed to other parts of the country, was based on accessibility: all the investigators worked in that area, and the SDCC, a governmental tertiary institution serving children with serious neurodevelopmental disabilities, is located there. Included in the capital area of Reykjavik are 17 PHCs. The plan for the screening project was introduced to the administrators of the Development Center for Primary Healthcare in Iceland and of the Primary Healthcare in the Capital Area, who all expressed their willingness to participate^13^. Description of the screening project was published in our previous studies^13,14^ and a summary is given later in this section.

Cluster randomization was used with the PHC as the unit of randomization. Altogether nine PHCs were randomly selected to participate in the screening project by an independent person in the presence of the investigators by drawing lots with their names. Following introductory meetings at each of these nine PHCs, their directors all gave their consent to participate in the project. Eight PHCs received usual care and constituted control group 1. A total of 4714 children in the target population were living in the capital area and were registered at the 17 PHCs that were randomized. Of them, 2531 children were assigned to be invited to screening, called the invited group, and 2183 children were assigned to control group 1. PHCs outside the capital area were without randomization; there, 2459 children were registered and were assigned to control group 2.

No child in the target population was excluded from the study as no child had been diagnosed with ASD before the start of the screening trial on March 1, 2016, according to the files of the SDCC. Thus, all children living in Iceland and registered for their 30-month well-child visits at a PHC during the period from March 1, 2016, to October 31, 2017, were included in the study.

For this study, a nationwide database on ASD diagnoses was kept at the SDCC. Using this database, children in the target population were followed-up from March 1, 2016, the beginning of the screening, to identify cases. The closing date for follow-up was October 31, 2019, when the children were between 54 and 79 months of age. The investigators scrutinized the records at the Center for Child Development and Behavior, the Department of Child and Adolescents Psychiatry at the Landspitali University Hospital, the Pediatric Department of Akureyri Hospital, and the Social Insurance Administration, and did not find children diagnosed with ASD, within the age range included in the study, which had been the case in a recent study of older children in Iceland^15^, indicating that there were no missing cases. Thus, only cases of ASD occurring in the SDCC database were included.

The healthcare system in Iceland is state-centered, mostly publicly funded, and with universal coverage. The following sections describe the content of the usual care. PHCs throughout the country offer a broad range of primary care services, including well-child care. After initial home visits by a nurse or a midwife in the first six weeks after birth, young children attend their neighborhood PHC 11 times until they start elementary school. During these visits, they receive developmental surveillance and broadband developmental screening as well as participate in a comprehensive vaccination program with over 95% participation rate^16,17^. The developmental screening instruments include the Parents’ Evaluation of Developmental Status^18^ which is administered at 12 and 18 months of age, accompanied by the Brigance Early Childhood Screen II^19^ at 30 and 48 months of age^17^.

The child’s first contact with the educational system is with the preschool service (nursery school), which children usually enter between ages 1 and 2 years, and where the goal is to monitor and promote the development of the children in close cooperation with the parents^20^. In 2018, 40% of the personnel in the preschools had a university degree in preschool teaching or other comparable education, including degrees or courses in the development and learning psychology of young children. The proportion of children in the population attending preschool at the age of 1 year is 48%, and 95–97% at the age of 2–5 years. At the preschool, approximately 10% of the children receive special support due to disability or social or emotional difficulties^21^. Education is compulsory for children aged 6–16 years and includes accommodation for special educational needs^22^. Social services provide financial support to parents of children with serious developmental disabilities or long-term illnesses, based on a valid medical certificate^23^.

If concerns of developmental disabilities are raised by parents or by professionals in the health care- or educational systems, this can, in collaboration with the parents, lead to preliminary assessment and subsequent referral to the SDCC for diagnosis. The SDCC provides each child with an interdisciplinary assessment, including a physical and a neurological examination by a pediatrician, an evaluation of autism symptoms by a psychologist using at least one standardized diagnostic instrument, and an interview with the parents by a social worker. Cognitive tests are administered at either the secondary or tertiary level of services. The interdisciplinary team reaches a consensus on the clinical diagnoses. If a clinical diagnosis is warranted, it is given based on the ICD-10 diagnostic system^24^.

In the present study, instruments used during the diagnostic process included the Autism Diagnostic Observation Schedule, Second Edition (ADOS-2; Modules 1 and 2)^25^ for assessment of autism symptoms, administrated by reliable clinicians; the Icelandic standardization of the Wechsler Preschool and Primary Scale of Intelligence, Revised (WPPSI-RIS) for assessment of cognitive ability^26^; and the Bayley Scales of Infant and Toddler Development, Third Edition^27^ for children who were not able to complete the WPPSI-RIS.

The screening program for ASD was implemented in the invited group (*n* = 2531), of which 1586 children were screened with the M-CHAT-R/F, as reported in previous studies^13,14^. The M-CHAT-R/F is a two-stage parent-report screening instrument designed to identify 16–30-month-old children who should receive a more comprehensive assessment^4,8^. An Icelandic translation and cultural adaptation of the instrument was used, and sensitivity in the sample of the population of 30-months-old children was 0.62, specificity 0.99, positive predictive value 0.72, and negative predictive value 0.99^14^.

The main outcome of the study is the rate of ASD according to the nationwide database at the SDCC. We also describe sex, origin of parents, age at referral, age at diagnosis, ADOS-2 comparison score, IQ/DQ verbal and performance scores among cases of ASD.

The occurrence of ASD cases during the follow-up from June 15, 2017, when the first child was diagnosed, until October 31, 2019, was measured by cumulative incidence during a 27-and-a-half-month period. The analysis was conducted in accordance with the intention-to-screen principle. For the calculation, the numerator was children diagnosed with ASD in the population and in the corresponding groups, while the denominator was all children registered for their 30-month well-child visits at the PHCs during the period from March 1, 2016, to October 31, 2017, and the number of children in the corresponding groups. Rate ratios were calculated with 95% confidence intervals (CIs). The rates were calculated as cumulative incidence (ASD/100 children), and exact rate ratio estimates, confidence limits, and *p*-values (two-tailed) were calculated using the Martin and Austin method^28^. The analyses were performed in Epi Info™.

Regarding ethical approval and consent, the study was conducted in accordance with the guidelines of the Declaration of Helsinki, approved by the Icelandic Data Protection Authority, the National Bioethics Committee of Iceland (VSNb2015110029/03.01; License Date: January 12, 2016), the Scientific Committee of the Healthcare of the Capital Area, and the University of Iceland (License Date: November 11, 2015), and the Scientific Committee at the SDCC (License Date: November 25, 2015). Written informed consent was obtained from the parents of all children who underwent screening; however, informed consent was not necessary from parents of the children who did not participate in the screening, or belonged to the control groups, since the study of these children was solely data-based, according to the approvals.

## Results

Altogether 119 children of the entire study population were diagnosed with ASD during the period from June 15, 2017, until October 31, 2019. The 2531 children in the invited group were offered screening^14^, and of these 1586 children participated, while 945 did not participate. The children who participated in the screening and screened positive were referred for assessment and in this way 18 children were diagnosed with ASD. Of the children who screened negative 11 were subsequently diagnosed with ASD. Among the children who did not participate in the screening 23 children were diagnosed with ASD. At the start of the screening two children were already suspected to have ASD and were later diagnosed with ASD, and this adds up to 54 children with ASD in the invited group. The children in the control groups received usual care; 40 children in control group 1 were diagnosed with ASD and 25 children in control group 2. Of the 119 children with ASD, 98 were male (82.4%) and 21 were female (17.6%), with a male-to-female ratio of 4.7:1. Eighty children (67.2%) had both parents of Icelandic origin and 16 children (13.5%) had one parent of Icelandic origin. The rest of the children had both parents of non-Icelandic origin (*n* = 23; 19.3%).

**Figure 1:**
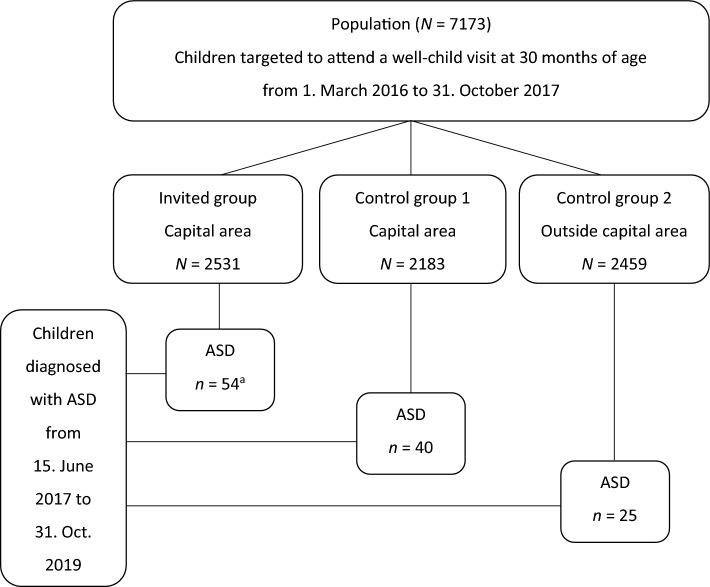
Children diagnosed with ASD in a group invited to a screening program and in two control groups. The flowchart shows the study population that included all children in Iceland who were registered at primary healthcare centers and were targeted to attend a routine well-child visit at 30 months of age from March 1, 2016, to October 31, 2017. Also, the number of children in each of the study groups, i.e., the group invited to the screening program during the above-mentioned period and the control groups who received usual care. Children in the invited group and control group 1 were registered at PHCs in the capital area of Reykjavik that were a part of the cluster randomization. Children in control group 2 were registered at PHCs that were outside the capital area and were not a part of the randomization. Finally, the number of children in each group who were diagnosed with ASD, according to a nationwide ASD registry, from June 15, 2017, when the first child was diagnosed, and to the end of the follow-up period on October 31, 2019. ASD, autism spectrum disorder. ^a^True-positive = 18, false-negative = 11, did not participate in the screening = 23, identified with concerns before screening = 2.

Figure 1 shows how the 119 ASD cases were divided into each of the three groups: the invited group, control group 1, and control group 2. Some of the children diagnosed in the invited group, altogether 29 children (18 true-positive and 11 false-negative), participated in the screening project during their 30-month well-child visit^14^. In addition, two children had been excluded from the previous screening study since developmental concerns had already been raised and referrals for diagnostic assessment were being prepared^13^. These children were included in the present study since they met criteria for diagnosis during the above-mentioned period. The rest of the children in the invited group (*n* = 23) did not receive autism-specific screening since their parents did not consent to participate in the screening study, had not attended the 30-month well-child visit at the participating PHCs during the inclusion period, or failed to receive an invitation to participate in the screening^14^.

The overall cumulative incidence of ASD was 1.66 (95% CI: 1.37, 1.99), as shown in Table 1 alongside the cumulative incidence for the invited group and the control groups. The rate was highest in the invited group and lowest in control group 2. The comparison of the rate of ASD in the invited group with the rates in the combined control groups, control group 1, and control group 2, are shown in Table 2 by rate ratio with the corresponding 95% CI. The rate ratio of the invited group versus the combined control groups was 1.52 (95% CI: 1.06, 2.19); the rate ratio of invited group versus control group 1 was 1.16 (95% CI: 0.77, 1.75); and the rate ratio of invited group versus control group 2 was 2.10 (95% CI: 1.31, 3.37).

**Table 1:** Number of cases, cumulative incidence per 100 of autism spectrum disorder, and 95% CI in the population, the invited group, the combined control groups, control group 1, and control group 2.

Population/groups	Denominator	Cases	Rate per 100	95% CI, lower/upper
Population	7173	119	1.66	1.37–1.99
Invited group	2531	54	2.13	1.60–2.78
Combined control groups	4642	65	1.40	1.08–1.79
Control group 1	2183	40	1.83	1.31–2.50
Control group 2	2459	25	1.02	0.66–1.50

*CI* confidence interval.

**Table 2:** Comparison of the invited group and the control groups, rate ratio, 95% CI, and *p*-value.

Groups and combination	Rate ratio	95% CI, lower/upper	*p*
Invited group versus combined control groups	1.52	1.06–2.19	0.024
Invited group versus control group 1	1.16	0.77–1.75	0.469
Invited group versus control group 2	2.10	1.31–3.37	0.002

*CI* confidence interval.

The clinical characteristics of the ASD cases in the groups are shown in Table 3. The proportion of males was highest in the invited group, and lowest in control group 1. Age at referral to the SDCC for diagnostic assessment and age at diagnosis were similar in all groups. However, the age at referral was lowest in the invited group, second lowest in control group 1, and highest in control group 2. The differences in age at referral were small, approximately one month or less.

**Table 3:** Clinical characteristics of autism spectrum disorder cases in the group invited for screening and in the two control groups.

Characteristics	Invited group (*n* = 54)		Control group 1 (*n* = 40)		Control group 2 (*n* = 25)	
	*n* (%)	*M* (*SD*)	*n* (%)	*M* (*SD*)	*n* (%)	*M* (*SD*)
Male	47 (87.0)		31 (77.5)		20 (80.0)	
Both parents of Icelandic origin	39 (72.2)		21 (52.5)		20 (80.0)	
Age at referral (months)		36.15 (8.16)		37.13 (6.93)		38.52 (9.54)
Age at diagnosis (months)		55.44 (8.27)		55.25 (7.81)		57.04 (8.90)
ADOS-2 comparison score		5.69 (1.56)		5.68 (1.88)		5.30 (1.87)
IQ/DQ verbal		66.05 (28.02)		59.11 (21.81)		62.15 (17.87)
IQ/DQ performance		85.29 (19.38)		79.20 (20.22)		80.56 (21.18)

*ADOS-2* Autism Diagnostic Schedule, Second Edition, *IQ/DQ* intelligence quotient/developmental quotient, *M* mean, *SD* standard deviation.

## Discussion

Our study compared the rates of ASD detected in a group invited to a screening program with the rates of ASD detected in two groups that received usual care. The invited group had a higher rate of ASD than the combined control groups, but that was only evident in the comparison with control group 2. The comparison with control group 1 yielded an elevated rate ratio, but with a wide 95% CI, which included one. Cluster randomization of children with PHCs as the unit of randomization enabled us to compare the invited group to control group 1. These groups were considered comparable in terms of cultural and social status and were determined to have equal access to specialized developmental services. The comparison between the invited group and control group 1 was the most important. Based on that comparison, the screening did not have a clear impact on the detection of ASD. In addition, we found that children in the invited group were not referred for diagnostic assessment at a younger age than children in each of the control groups, nor did they receive their ASD diagnosis earlier.

The overall cumulative incidence of ASD for the three groups was 1.66%. This result is higher than in a recent systematic review where the overall median global prevalence of ASD was estimated to be 1%^29^. Studies that have investigated prevalence or cumulative incidence by age found that it ranged from 0.37 to 1.56% among children 4 years of age^30–32^.

We are not aware of other published studies comparing a group invited to screening to an external comparable control group using cluster randomization for the purpose of evaluating whether ASD was detected earlier and at higher rates in an invited group than in a control group. The United States Preventive Services Task Force has not recommended screening for ASD in the general pediatric population because of lack of evidence from studies comparing a screened group with a group receiving usual care indicating that treatment starting at a younger age improves outcomes measured with standardized tests for symptom severity and cognitive functioning^33^. To evaluate screening for ASD, such a study is planned and intends to screen for ASD and administer high-quality treatment with long term follow-up of outcomes^12^.

Amongst the strengths of the present study is that it was population-based: the main comparison (between the invited group and the control group 1) was between groups that share a common cultural and social background as well as equal proximity to the institution responsible for the diagnosis of the cases. The assignment to the groups was based on cluster randomization. The ASD diagnoses were received from a nationwide database of ASD cases, the diagnostic procedure was based on interdisciplinary teamwork, and the use of a gold standard diagnostic instrument.

This study has some limitations. Partly due to privacy protection issues, it was not possible to follow an individual child in each group from the start of the screening to the diagnosis of ASD or to the end of follow-up. However, the age at referral and the age at diagnosis were similar in the study groups, and the cases had similar clinical features. Because of the similarity of age at referral and age at diagnosis, it was considered unnecessary to test different lengths of follow-up. In addition, it was not practical or possible to blind the clinical personnel at the SDCC ascertaining the diagnoses. The rationale for using cluster randomization with PHCs in the capital area as the unit of randomization was mainly the accessibility, and that may have diminished the risk of contamination of the control groups. However, we cannot be sure that the reported gains resulting from the educational course on ASD, held for clinicians serving children in the invited group^13^, did not contaminate the control groups, particularly in the capital area, for reasons such as temporary rotations of staff between different PHCs. The comparability between the invited group and control group 2 was hampered not only by the fact that control group 2 included rural areas and does not have the same access to care as the groups in the capital area, but also because of a difference in educational levels. Several studies have found that the higher the prevalence of ASD, the greater the level of urbanicity^34–37^, and that a higher educational level of parents is associated with ASD^34^. We did not collect data on parental education. However, a study in Iceland found that university education was twice as common in the capital area as in other parts of the country^38^. The lower rate of ASD in the rural area (control group 2) than in the urban area (control group 1) calls for improved access to developmental services in rural areas. The study base was the entire population of children in Iceland and, with the definition of the inclusions criteria, framed the size of the study. By extending the inclusion period we would have obtained larger groups. However, simultaneously that may have introduced time-trend effects in the detection of ASD.

Similarly designed studies, comparing groups invited to screening to external control groups, are needed to explore whether screening detects ASD earlier than usual care, as the present study may be considered not large enough. The question remains as to whether children with ASD detected through screening will have better long-term outcomes because of early intervention than children detected through the usual care.

## Conclusion

The invited group and control group 1 were considered comparable in terms of cultural and social status, access to specialized services, and proximity to the institution responsible for the ASD diagnoses. The children were assigned to groups via cluster randomization, where the unit of randomization was the PHC. The rate of ASD was higher in the invited group than in the control groups; however, interpreting the results is difficult because of the wide confidence intervals. So, one cannot firmly conclude from this study that the screening program detected ASD more readily than did the usual care.

## Data Availability

The data presented in this study are available from the corresponding author upon request, dependent on permission from the Data protection Authority and the National Bioethics Committee of Iceland.
